# Dr. Balfour in Reply to the Review, in No. 212 of This Journal, of His Treatise on Rheumatism

**Published:** 1817-03

**Authors:** 


*For the London Medical and Physical Journal.*

### Dr. BALFOUR *in Reply to the Review, in No. 212 of this Journal, of his Treatise on Rheumatism.*

IF self-defence is justifiable and necessary in any case, it must be in that of an author when his works are criticised "in the determined spirit of cavil." Having received from some of the most eminent medical practitioners, and, indeed, from people of every rank and denomination, in this city, the most honourable testimony to the value of my improvements in the cure of rheumatism; it is painful to be laid, as I am, under an imperious necessity to rebuff a critique, which represents my claims to novelty as arrogance, my my statement of facts to be exaggeration. I must, therefore, tell my Reviewer, in the outset, that a tissue of palpable inconsistency, unwarranted assertion, ungentlemanlike repulsive sneers, and illiberal inuendos, can never be sustained as "a candid statement of the principles and practice" of any book.

In the first place, then, the language my Reviewer holds concerning the principles and practice of my book, is the very reverse of that employed by another gentleman, in a review of the same principles and practice, in No. 197 of this same Journal. There my practical remarks are called valuable; my Essay, truly valuable; so much so, that the value of my improved practice would not be lessened, the writer thinks, whatever the theory of rheumatism might turn out to be. And, he not only gives me full credit for my improvement, but thinks it right to state, that my practice is adopted in London, with great advantage. The Reviewer, however, with whom I have at present to deal, denies novelty to both my theory and practice; represents my statements as illusory; and cautions his readers against giving them implicit credit. Opinions, indeed, more irreconcilably hostile than those of the two Reviewers of my Treatise on Rheumatisms, are not to be found in the annals of criticism. "But had they (the two critiques) been the production of the same writer, we see no reason, (you say,) why further experience should not have altered his opinion."—*Lond. Med. and Physic. Journ. No.* 214. Unfortunately, however, for the correctness of your opinion, and the consistency of your Journal, in this instance, the first Reviewer had experience of my practice, and bestows on it the highest praise; the last Reviewer had no pretensions to experience on the subject. And, as the spirit in which his paper is written indicates no extremely tender feelings on the part of its author, could he have told me that my plan of curing rheumatism had been unsuccessful in the hands of other practitioners, he would not have spared me the mortification. But, to determine this point, one question only is necessary: Could a man consistently affirm at one time, that a certain practice is valuable—truly valuable, an improvement, and adopted in London with great advantage; and, at another time, that the same practice is not new, that it is illusory, that its details should be read by those only who are on their guard against exaggeration, and that it has not been put to the test by any but the author? If you can answer this question in the affirmative, I have no more to say to you on this or any other subject. What is an improvement, must be new; what is truly valuable, cannot be an illusion; and, concerning concerning what is already adopted in London with great advantage, it is preposterous to be at a loss to determine "Whether it is likely to be attended with general success in the hands of individuals who have no system to support, and no pre-conceived notions to ratify."

In the second place, "We must be permitted (says the Reviewer) to say, that Dr. Balfour, in contending against those theories of rheumatism which make it consist in lentor of the fluids, or a peculiar acrimony,—displays his ingenuity to very little purpose." The only mention made of these hypotheses, in all my Treatise, is in these words: "Dr. Macbride and others imagine it to consist in a peculiar acrimony; others, in a lentor of the fluids."—P. 2. Now, the bare mention of hypotheses as having been once mentioned, can never be construed into strenuous endeavours to refute these hypotheses. The Reviewer, therefore, is guilty, in this instance, of a wilful misrepresentation; and with no other possible view, than to represent me as ignorant of the opinions at present entertained concerning the essential nature of the disease of which I treat. Most critics confine their observations to what is really recorded in the books they review; this Reviewer attributes a fault to me, which is not to be found in my treatise, and which never entered into my contemplation.

In the third place, "Nor is the notion quite new, (says the Reviewer,) that the seat of rheumatism is membranous rather than muscular." That rheumatism has its seat, most commonly, in membranous parts, is obvious to the most superficial observer. But I first advanced the opinion, that rheumatism has its seat in the cellular membrane, when it attacks the belly of a muscle or even a single muscular fibre equally, as when it attacks a capsular ligament, or the sheath of a tendon. Therefore my theory is new.

In the fourth place, "It may not be improper, (continues the Reviewer) to make the same observations in respect to our author's claims to practical novelty that we have before advanced in respect to his theorizing; but, surely friction and other mechanical stimuli had been thought of and practised long before our author favoured the world with his present treatise." That friction is coeval with rheumatism, no person in his senses, I believe, will deny; and it will be difficult for the Reviewer to point out a sentence in my book, where the contrary is mentioned. My mode of applying friction, however, is not the less new on these accounts. Suppose my Reviewer and I were called to two patients in exactly similar circumstances, "lying on their bed under the pains of rheumatism," unable to move their arms on account account of a few fibres only, which often happens, of their *deltoids* being affected. Suppose, further, that we applied friction to our patients; my Reviewer in the usual way, I in my way. He would make little or no impression on his patient for a great length of time, if ever; I would ease mine in five minutes. He would require the aid of "cinchona, and of ammoniated tincture of guaiacum in large quantities," and, perhaps, of "galvanism" too, to ease his patient; my patient would require no medicine. Now, my Reviewer himself must admit, that there is a difference betwixt friction in the usual way, and as applied by me. It consists in this: knowing, at least believing, that the pain, in such cases as I have supposed, arises from pressure on the nerves by preternaturally distended capillaries; I plunge the points of my fingers at once into the belly of the muscle, directly upon the pained part; compressing and rubbing it till the pain subsides or flies off altogether; and, when I remove my fingers, motion is found practicable. I now call the neighbouring parts into action by percussion, and then support the whole with a bandage where applicable. "I directed the patient to make diligent use of friction as often as he removed the bandage; but not of that kind of friction only which consists in stimulating the vessels of the skin. The pains in this case were deep-seated among the bones, and demanded a species of friction calculated to promote circulation in the extreme vessels of the parts affected."—See my *Treatise on Rheumatism*, page 74. Now, I call upon my Reviewer to say, when, and by whom, friction was ever applied on such principles, in such a manner, and with such effect, in rheumatism, before I did it. Till he does this, I am justified in denominating my plan, an entirely new mode of treating the complaint.

In the fifth place, with regard to the reader being "now and then reminded of Perkins's tractors and animal magnetism, while perusing (my) statements,"—I must inform the Reviewer, that, if he will take the trouble to look into Nos. 199 and 203, of the London Medical and Physical Journal, he will find cases of acute rheumatism treated avowedly on my plan, with a degree and celerity of success not exceeded by any fact recorded by me in all my thirty-three cases; and, by gentlemen too, "who have no system to support, and no pre-conceived notions to ratify." The candour of these gentlemen is, indeed, a perfect contrast to the illiberal inuendos of this Reviewer. "They (bandages) are not like medicines exhibited internally, concerning whose operation and effects we are liable to form the most erroneous conclusions. Their effects are immediate and visible. We are not not left at a loss to determine, whether the good produced is to be ascribed to the efforts of nature, or to the remedy employed; nor is it a matter of doubt, whether the remedy is of real advantage or not? There can be no uncertainty as to the powers of a remedy, which, the moment it is applied, enables a person to walk, who immediately, and for many weeks before, could not set the sole of his foot to the ground."— See my *Treatise on Rheumatism*, pages 68 and 69. If my patients were under illusion, at the time of my attendance, and only imagined themselves better, it is very surprising they should have continued in the same state of body and mind ever since. Madame Rey, (case 14,) for instance, who, when I was called to her, was unable to rise off her chair, had little or no use of her hands, and had not walked a step for eight years, undertook a journey, this last summer, from Edinburgh to the south of France!—Mr. Grant, (case 21,) aged 80, walked on crutches, with great difficulty, eighteen months ago; but never used them after my first visit. I met him in the street the other day, perfectly free from complaint. If this is all illusion, then Perkins's tractors are, in point of deception, a mere mock to friction, percussion, and bandages!

In the sixth place, of all the observations of the Reviewer, the most insufferable to an ingenious mind is the following: "In this view we may remark, that the perusal of the work before us may prove useful to all such as know how to add the *granum salis* to the reports of authors who are advocating a favourite hypothesis." The low and ungenerous suspicion of my having accommodated facts to hypothesis, can be entertained by that man only who could *make* a case to support a theory. I have advanced a theory, to be sure,— as likely to be true as those that have gone before it. But should the theory of rheumatism be "solved in a manner contrary to Dr. Balfour's opinion, that would not lessen the value of his improved practice." *London Med. and Phys. Journal*, No. 197.—What advantage, I would ask the Reviewer, could I have proposed to myself, in giving to the world a string of exaggerated statements. Money could not have been my object, for I have no nostrum to vend. The Reviewer himself admits, that thirty pence would purchase all the medicines I prescribed in thirty-three cases. And ephemeral, indeed, would that reputation have been, which I could have acquired by imposture. Aware, from the opposition which I experienced before I published my treatise, that it would be criticised "in the determined spirit of cavil," I read to all my patients within my reach their respective cases, lest I should, in my zeal, have unintentionally given the slightest colouring to any of them. Could man shew more regard for truth, respect for his own credit, or deference to public opinion? More than this,—Dr. Wood, of Perth, witnessed the effects of my operations on his patient, Mr. Chalmers; Dr. Gregory, and Mr. Bryce—President of the College of Surgeons, met me almost every day during my attendance on Lord Meadowbank, who revised his own case, and who was not the man to have complimented any human being at the expence of one jot of the truth.

I have been thus circumstantial in my reply, out of respect to those who have honoured me with their confidence, and unqualified approbation of my practice in rheumatism.

*Edinburgh; Jan.* 7, 1817.

---

A considerable part of the above paper containing original matter, and the remainder, for the most part, authorities in the writer's favour, we have thought it fair to insert it, with no other curtailments than Dr. Balfour has permitted. We may state, that, whilst our reviewers have given such general satisfaction, few authors have had courage to object to them. We must, therefore, shew our impartiality in the present instance. For the future, however, we give notice that, if answers of any considerable length are sent us, unless they contain much original matter, we must decline inserting them. They may, indeed, be circulated with our Journal, if the author is willing to pay the expence of paper and printing. But our correspondents and readers will be ill requited for their favours and indulgencies, if our numbers are to be filled with the complaints of authors.—EDIT.

---

*For the London Medical and Physical Journal.*

